# The Gray Matter Volume of the Amygdala Is Correlated with the Perception of Melodic Intervals: A Voxel-Based Morphometry Study

**DOI:** 10.1371/journal.pone.0099889

**Published:** 2014-06-12

**Authors:** Xueting Li, Alain De Beuckelaer, Jiahui Guo, Feilong Ma, Miao Xu, Jia Liu

**Affiliations:** 1 Department of Psychology, Renmin University of China, Beijing, China; 2 Department of Personnel Management and Work and Organizational Psychology, Ghent University, Ghent, Belgium; 3 Institute for Management Research, Radboud University Nijmegen, Nijmegen, The Netherlands; 4 School of Psychology, Beijing Normal University, Beijing, China; 5 Center for Collaboration and Innovation in Brain and Learning Sciences, Beijing Normal University, Beijing, China; UNLV, United States of America

## Abstract

Music is not simply a series of organized pitches, rhythms, and timbres, it is capable of evoking emotions. In the present study, voxel-based morphometry (VBM) was employed to explore the neural basis that may link music to emotion. To do this, we identified the neuroanatomical correlates of the ability to extract pitch interval size in a music segment (i.e., interval perception) in a large population of healthy young adults (*N* = 264). Behaviorally, we found that interval perception was correlated with daily emotional experiences, indicating the intrinsic link between music and emotion. Neurally, and as expected, we found that interval perception was positively correlated with the gray matter volume (GMV) of the bilateral temporal cortex. More important, a larger GMV of the bilateral amygdala was associated with better interval perception, suggesting that the amygdala, which is the neural substrate of emotional processing, is also involved in music processing. In sum, our study provides one of first neuroanatomical evidence on the association between the amygdala and music, which contributes to our understanding of exactly how music evokes emotional responses.

## Introduction

*“Without music, life would be a mistake.”*

— *Friedrich Nietzsche, Twilight of the Idols*

Music is a powerful tool for evoking emotional responses. Tears burst out uncontrolledly when “The Queen's Epicedium” (Henry Purcell and John Blow, 1695) is performed, whereas intense joy floods our heart when we hear “The Blue Danube” (Johann Strauss II, 1866). Indeed, behavioral studies have shown that music evokes various emotions [1], [2], and can help patients feel less anxious and reduce postoperative pain [3]–[5]. However, little is known about the neural basis that links music and emotion. In the present study, we addressed this question by exploring the neuroanatomical correlates of individuals' behavioral performance in music processing.

The amygdala, a core component in the neural circuits of emotional processing and emotional experiences [6], [7], has attracted attention for its significance in processing music. Previous functional neuroimaging studies have demonstrated the amygdala's involvement in music processing. First, exposure to music scoring high on emotional valence (e.g., pleasant and unpleasant music) activates the amygdala. Specifically, this region of the brain is activated when participants experience a positive emotion after being exposed to pleasant music [8]–[10] or when they experience a negative emotion after listening to unpleasant music [8], [10]–[13]. Second, in addition to responding to pleasant or unpleasant music, the amygdala may respond to the occurrence of fairly abstract musical features, such as unexpected chords (the processing of which is perceived as being less pleasant than the processing of expected chords) [14].

In contrast, structural magnetic resonance imaging (MRI) studies relying on the measurement of cortical thickness [15]–[17] and/or voxel-based morphometry (VBM) [15], [16], [18]–[20] have only focused on frontotemporal circuits as neuroanatomical correlates of music processing. While the role of the amygdala in music processing is largely ignored in structural MRI studies, there is abundant evidence showing that the anatomical structure of the amygdala is correlated with emotional processing. For example, amygdala gray matter volume (GMV) or density is correlated with magnitude of stress [21], [22] and anxiety [23] in the normal population, and the change of amygdala volume is a neural signature of a variety of emotion-related disorders, such as major depressive disorder [24]–[27], bipolar disorder [28]–[30], borderline personality disorder [31], [32], posttraumatic stress disorder [33], and autism [34]. Finally, lesions of the amygdala severely impair emotional processing, such as emotion recognition [35]–[38], emotion arousal [39], and emotion judgment [40].

In contrast to the well-established link between the amygdala and emotion, the failure to find the association between the anatomical structure of the amygdala and music processing may be the consequence of some critical study characteristics. First, previous studies did not focus on the amygdala or other subcortical regions (at least they did not report significant results pertaining to these regions). Second, the music tasks relied on in previous studies focused on certain musical properties, but these studies did not explicitly examine whether these musical properties were linked to emotion (at least they did not report significant statistical results attesting to such links). Finally, these studies used small sample sizes (typically less than 100 subjects), resulting in a low probability for detecting the neuroanatomical correlates of music processing (i.e., low statistical power) [41].

To investigate whether the GMV of the amygdala is associated with music processing, we measured participants' ability to extract pitch interval size (i.e., interval perception), a core ability of music processing [18], [42]. After validating that interval perception was correlated with daily emotional experiences, we examined correlations between participants' amygdala GMV and interval perception. Unlike the abovementioned studies that used small participant populations, our study examined a larger sample of participants (*N* = 264) to provide more detailed information regarding the neuroanatomical correlates of interval perception of an individual. Based on previous functional MRI studies that indicate that the amygdala is involved in music processing, we predicted that participants with better interval perception would have a larger amygdala.

## Materials and Methods

### Ethics Statement

Both behavior and MRI protocols were approved by the Institutional Review Board of Beijing Normal University (BNU). Written informed consent was obtained from all participants prior to the experiment.

### Participants

Two hundred and seventy college students (160 females; age: *M* = 21.9 years, standard deviation (*SD*)  = 0.9 years) from BNU, Beijing, China, participated in the present study. Participants had not received any formal music training at the time of testing, and reported no psychiatric illness, hearing impairment, neurological disorder (e.g., epilepsy, traumatic brain injury, neurodegenerative disorders, or cerebrovascular disease), or mental retardation.

The participants completed a battery of tests consisting of music, language, personality, and general intelligence measures. The whole test battery took about 2 hours to complete, and the tests reported here took about 45 minutes in total. The tests that addressed language ability are not reported here.

### Measurement of interval perception

Interval perception was assessed using an interval task that measured an individual's ability to extract pitch interval size. The task was a modified version of the interval subtest of the Montreal Battery of Evaluation of Amusia (MBEA) [43]. The modified version of the interval subtest of the MBEA was designed to maximize the individual differences in interval perception, which was based on a pilot study with another set of participants (*N* = 40) to test the discriminative power of the 30 experimental trials included in the original test. The discriminative power of each trial was indexed by its accuracy. Because the chance level is 50%, the accuracy that has the highest discriminative power is 75%. The 20 experimental trials with an accuracy closest to 75% were included in the modified version, while the response type (“same” versus “different”) was balanced. The other 10 experimental trials were discarded because they failed to demonstrate relatively adequate levels of discriminative power (i.e., either ceiling or floor effects). The experiment trials in the modified version are listed in the Table S1 along with their accuracy.

**Figure 1:**
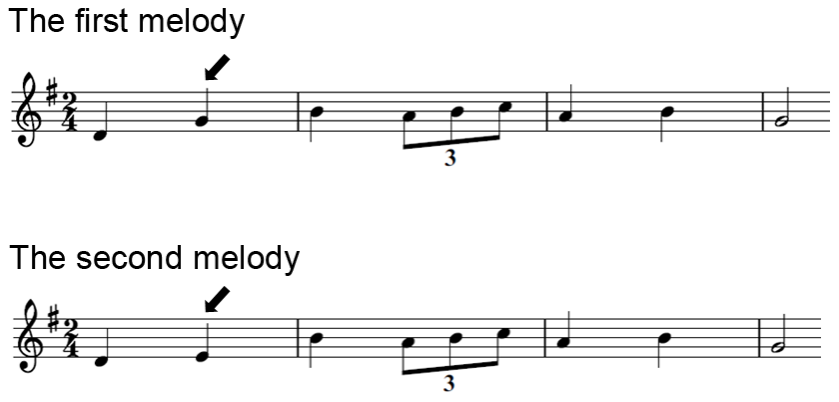
Exemplar stimuli in the interval test. Note that the second melody was nearly identical to the first melody, except that the second note of the second melody (see arrow pointing downwards) violated the pitch interval structure as indicated by the first melody.

In each task trial, participants were successively exposed to two melodies, both of which were composed according to the rules of the Western tonal system and were intentionally written with sufficient complexity to guarantee its processing as a meaningful structure rather than as a simple sequence of tones [43]. Melodies were presented as a synthetic piano sound generated with music production software (Reason, Stockholm, Sweden), which ranged from 7 to 21 notes, and lasted 4.0–6.4 s (*M* = 5.1 s). The interval between the two melodies was 2 s. Each pair of melodies was preceded by a warning tone. Half of the experimental trials presented two identical melodies (“same”), whereas the other half presented a second melody that was nearly identical to the first, except that a single note (randomly positioned) violated the pitch interval structure (“different”) (Fig. 1). These two trial types were randomly interleaved in the task.

Besides the experimental trials, there were two practice trials and one catch trial. Both the practice trials and catch trial were included in the original version of the interval subtest of the MBEA. The practice trials preceded the experimental trials, which were the same as the experimental trials except that they were presented with feedback. If the participant made a wrong judgment on a practice trial, a screen immediately displayed the message “wrong judgment.” When a participant judged incorrectly in one or both of the two trials, the instruction screen was again presented to the participant, followed by the presentation of the same two practice trials. This was repeated until the participant answered the two practice trials correctly. The catch trial was designed in such a way (1) that an attentive participant should make the correct judgment, and (2) that a melody with a meaningful musical structure was followed by a melody comprised of a simple sequence of music notes without musical structure. If the participant made the wrong judgment in the catch trial, his or her data were excluded from further analysis. The catch trial was randomly inserted in the sequence of the experimental trials.

During the task, participants sat in a comfortable chair in a quiet room and faced a computer screen positioned 80 cm from the participant. The participant was instructed to wear headphones through which melodies were presented. Before the test, a melody similar to the melodies used in the interval test was presented so that the participant could adjust the volume to the preferred level. A trial started with a warning tone, and then two melodies were played successively. Participants were instructed to judge whether the two melodies were the same or not. The instructions explicitly mentioned that differences between the pitch structures, if present, might be subtle. The participants recorded their judgment using a computer mouse to select the “same” or “different” button presented on the computer monitor. There was no time limit for the participant to make the judgment.

The participant's interval perception was indexed as the number of experimental trials with correct responses (i.e., same or different pitch structure). Therefore, the maximum attainable score was 20, with a higher score indicating better interval perception.

### Measurement of emotional experiences

To measure participants' daily emotional experiences, we used two emotion-related statements, “I experience a wide range of emotions and feelings” and “I rarely experience strong emotions” (reversed item), from the Big Five Personality Inventory (NEO Personality Inventory, Revised) [44]. Participants were asked to report the extent to which their daily emotional experiences matched each statement on a five-point Likert scale (0: strongly disagree; 4: strongly agree). The total score was used to index the participants' emotional experiences in daily life, with a higher score indicating stronger and more diverse emotional experiences.

### Measurement of general cognitive ability

Participants' general cognitive ability (i.e., IQ) was measured with Raven's Advanced Progressive Matrices [45]. Because the participants were quite homogenous (i.e., college students), IQ scores were calculated as the number of Raven items with correct responses. Therefore, the maximum score was 48, with a higher score indicating better general cognitive ability.

### MRI acquisition

Participants were scanned using a Siemens 3T-scanner (MAGENTOM Trio, a Tim system) with a 12-channel phased-array head coil. The scanner was housed at BNU Imaging Center for Brain Research, Beijing, China. MR structural images were acquired using a 3D magnetization-prepared rapid gradient-echo (MP-RAGE) T1-weighted sequence (TR/TE/TI = 2530/3.39/1100 ms, flip angle  = 7 degrees, FOV  = 256×256 mm). To cover the whole brain, 128 contiguous sagittal slices were acquired with 1×1 mm in-plane resolution and a 1.33 mm slab thickness.

### Image processing for VBM

VBM was used to explore the neuroanatomical correlates of interval perception [46]. VBM provides a quantitative measure of tissue volume for each voxel [46]. This analysis was performed using SPM8 (Statistical Parametric Mapping, Wellcome Department of Imaging Neuroscience, London, UK) on T1-weighted structural images. First, the quality of structural images was assessed manually by visual inspection. Second, the brain origin for each participant was manually set to the anterior commissure. Third, structural images were segmented into four distinct tissue classes: gray matter (GM), white matter, cerebrospinal fluid, and everything else (e.g., skull and scalp) using a unified segmentation approach [47]. Fourth, the GM images were rigidly aligned and resampled to 2×2×2 mm. Fifth, the GM images were nonlinearly registered using the Diffeomorphic Anatomical Registration through Exponential Lie algebra (DARTEL) registration method [48]. DARTEL registration involves repetitively computing the study-specific template and warping the GM images to the generated template. Sixth, the GM images were spatially normalized to the Montreal Neurological Institute (MNI) 152 space. Seventh, to preserve the original GMV for each participant, the normalized individual GM images were modulated by multiplying each voxel's GM value with the voxel-specific Jacobian determinants derived from the spatial normalization [49]. The modulated GM images, depicting a GMV measure for each voxel, were then smoothed using an 8-mm full-width at half-maximum (FWHM) isotropic Gaussian kernel. Finally, the modulated GM images were masked using an absolute masking with a threshold of 0.2. Thus, all voxels with a GMV value less than 0.2 were excluded. Statistical analysis was used to further examine the masked-modulated GM images.

### Statistical analysis

The statistical analysis was performed in two parts. First, a whole brain analysis was performed to explore all neuroanatomical correlates of individual differences in interval perception. Second, a region of interest (ROI) analysis was performed to further describe the relationship between GMV in the amygdala and interval perception.

#### Whole brain analysis

Voxel-by-voxel, a generalized linear model (GLM) was performed using the GMV extracted from the GM images as the dependent variable, with interval perception scores as the covariate of interest and sex and the total GMV of the brain as confounding covariates. A false discovery rate (FDR) using a threshold of *p*<0.05 was used to correct for multiple comparisons across the whole brain.

#### ROI Analysis

As previous functional MRI studies have confirmed the amygdala's role in music processing, the amygdala was selected as an ROI. We relied on the Harvard-Oxford subcortical probabilistic structural atlas [50] and used a probability threshold of 50% to define the left and right amygdala as two ROIs (anatomical label). For each participant, we averaged GMV across all voxels within each ROI. To describe the neuroanatomical correlates of interval perception, we performed partial correlations between the average GMV of the left and right amygdala and the interval perception score, while controlling for the effects of the total GMV of the brain and sex. To test the specificity of the correlation between amygdala GMV and interval perception, we performed partial correlations between the GMV of the left and right amygdala and interval perception, while controlling for the effects of IQ, the total GMV of the brain, and sex.

### Participant exclusion

Two participants (Male: 2; Female: 0) were excluded from the interval test. One participant did not follow the instructions, which was disclosed in the catch trial, and another participant's interval perception score was three SDs below the mean. Another eight participants (Male: 4; Female: 4) did not finish the questionnaire on their daily emotional experiences. Another four participants (Male: 2; Female: 2) were excluded the VBM analysis because of extraordinary scanner artifacts (e.g., head movement during MRI scanning) or abnormal brain structure (e.g., unusually large ventricles). Finally, one other participant (Male: 0; Female: 1) did not participate in the IQ test.

Therefore, the data from 260 out of 270 participants (156 females) were included in the correlational analysis on the relation between interval perception and daily emotional experiences. For the VBM analysis, data were available from a total of 264 participants (158 females). Finally, data from 263 participants (157 females) were used for testing the specificity of the association between interval perception and amygdala GMV by controlling for IQ.

## Results

A participant's interval perception was indexed as the number of correct responses in the interval test. The mean and *SD* of the scores were 16.37 and 2.59, respectively. An independent sample *t*-test revealed no statistical difference in interval perception (*t*(262)  = 1.14, *p* = 0.25) between males (*M* = 16.05; *SD* = 2.75) and females (*M* = 16.42; *SD* = 2.47). Importantly, participants who experienced stronger and more diverse emotions in daily life scored higher in the interval test (*r* = 0.20, *p* = 0.001), and this association remained even when participants' general cognitive ability and gender were regressed out (*r* = 0.18, *p* = 0.004). Therefore, the fact that interval perception was correlated with emotional experiences offers opportunities to examine the neural basis that may link music to emotion by exploring the neuroanatomical correlates of individual differences in interval perception.

**Figure 2:**
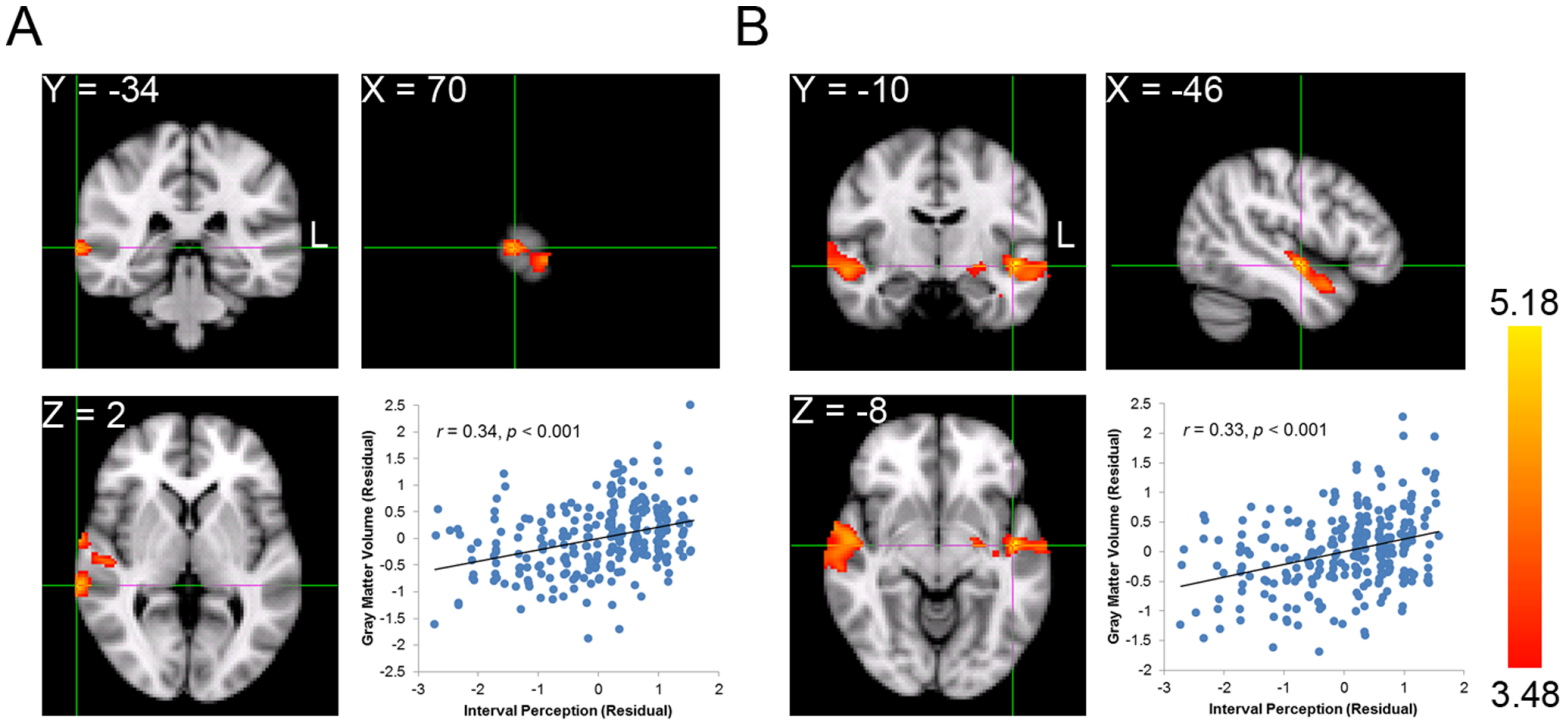
Neuroanatomical correlates of interval perception in the temporal cortex. *A.* The peak voxel in the right temporal cortex is located in the superior temporal gyrus. The scatter plot shows distributions of scores on interval perception vs. mean GMV of all the voxels in the cluster after controlling for sex and total GMV. Each dot represents one participant. *B*. The peak voxel in the left temporal cortex is located in the planum polare. The scatter plot shows distributions of scores on interval perception vs. mean of GMV of all the voxels in the cluster.

**Figure 3:**
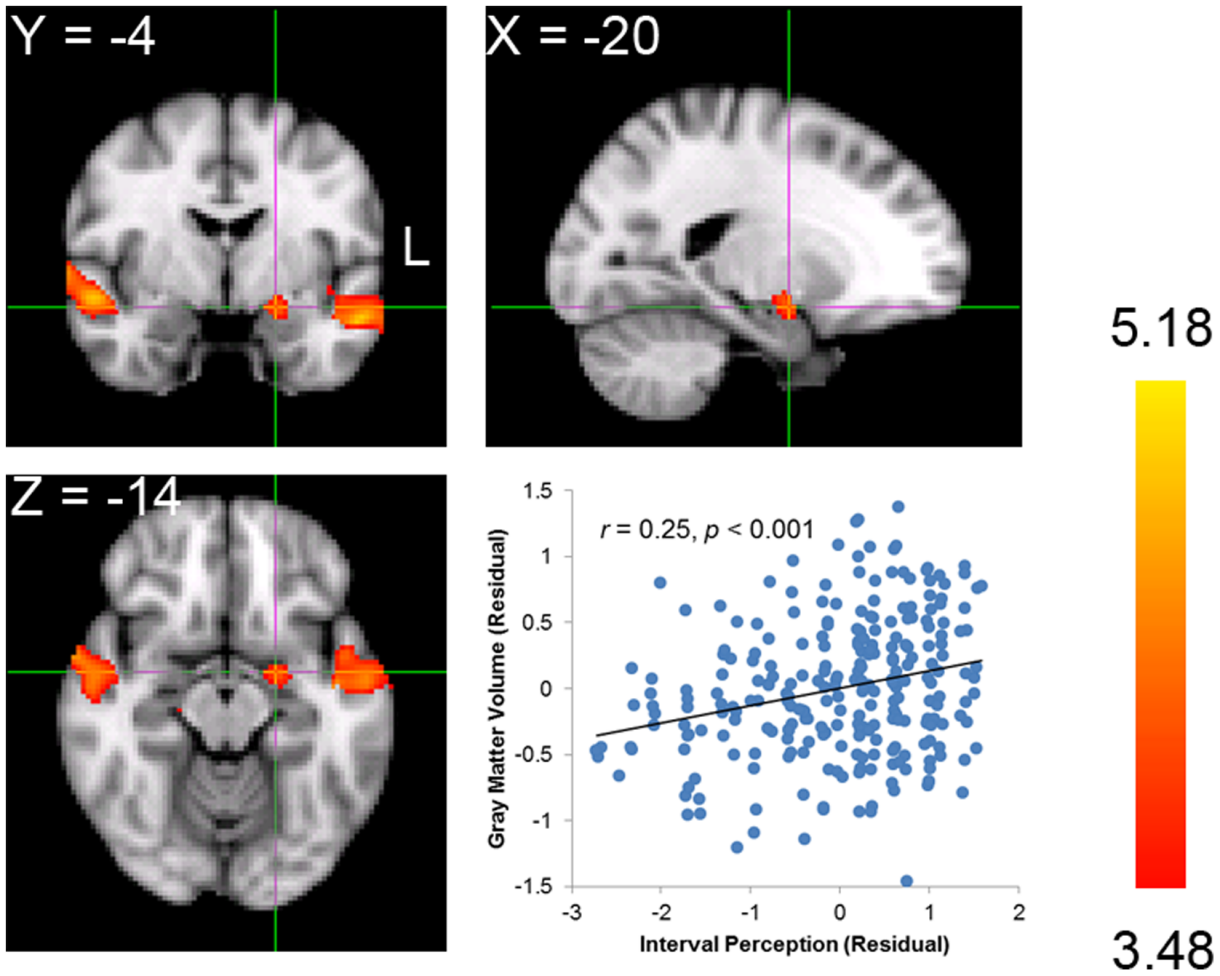
Neuroanatomical correlates of interval perception in the amygdala. The peak voxel is located in the left amygdala. The scatter plot shows distributions of scores on interval perception vs. mean GMV of all the voxels in the cluster after controlling for sex and total GMV. Each dot represents one participant.

To do this, we first correlated the GMV of each voxel across the whole brain with the interval perception for each participant (i.e., whole brain analysis). Significant positive correlations between interval perception and GMV were found in three voxel clusters (Table 1). The clusters in the left and right temporal cortex included portions of the superior temporal gyrus, middle temporal gyrus, planum polare, and Heschl's gyrus. The peak voxel of the cluster in the right temporal cortex was located in the superior temporal gyrus (MNI coordinate: 70, −34, 2; FDR-corrected *p*<0.05; Fig. 2A), whereas the peak voxel of the cluster in the left was in the planum polare (MNI coordinate: −46, −10, −8; FDR-corrected *p*<0.05; Fig. 2B). These clusters correspond with the clusters identified in previous studies that revealed that the auditory cortex participates in pitch processing [14], [16], [19]. More important, the third cluster was comprised of voxels corresponding to the left amygdala, indicating a significant positive correlation with interval perception (MNI coordinate: −20, −4, −14; FDR-corrected *p*<0.05; Fig. 3). This finding is consistent with our hypothesis that larger GMV in the amygdala is associated with better interval perception. Moreover, this finding is consistent with previous functional MRI and positron emission tomography (PET) studies [9], [11] that implicate the amygdala in music-related tasks.

**Table 1:** Clusters of voxels correlated with interval perception.

Brain Regions	Cluster Size (voxels)	Zscore	Correlation Coefficient	MNI coordinate		
				x	y	z
Right Superior Temporal Gyrus	1010	4.80	0.34	70	−34	2
Left Planum Polare	811	5.18	0.33	−46	−10	−8
Left Amygdala	91	4.11	0.25	−20	−4	−14

*Note*: MNI  =  Montreal Neurological Institute.

However, our results, based on the whole brain analysis, failed to reveal significant correlations between the right amygdala and interval perception. There are two possible explanations for this finding. Unlike the left amygdala, the right amygdala is not related to interval perception, or the right amygdala is indeed associated with interval perception, but significance levels did not surpass the strict critical probability levels determined by the FDR correction for multiple comparisons.

To obtain more detailed insight into the specific role of the amygdala, we performed an analysis in which the amygdala was defined as the ROI. Correlations between amygdala GMV and interval perception were computed while controlling for the effects of total GMV of the brain and sex. The ROI analyses revealed that the left and the right amygdala were similar, and both were related to an individual's interval perception (Left: *r* = 0.22, *p*<0.001; Right: *r* = 0.17, *p* = .007). Note that the magnitude of the correlations observed between interval perception and amygdala GMV were modest in this study. To estimate the number of participants required to replicate our study, we performed a power analysis with G*Power [51]. We found that with an estimate of *r* = 0.22, at least 159 participants were required to discover the correlation between interval perception and amygdala GMV at a power of 0.80. However, to discover the association between the temporal cortex and interval perception, only 65 participants were needed with an estimate of *r* = 0.34 (peak correlation coefficient of the cluster in the right temporal cortex, Table 1) at a power of 0.80. The power analysis demonstrates that previous studies with a smaller number of participants are sufficient to discover the temporal cortex, but are possibly underpowered to detect the amygdala in music processing (for a review, see [41]).

One may argue that the association observed was not specific to interval perception, because interval perception was also correlated with general cognitive ability (measured with Raven's Advanced Progressive Matrices, *M* = 37.1, *SD* = 4.04) (*r* = 0.24, *p*<0.001). To rule out this possibility, we tested whether the association remained after controlling for IQ. We found that interval perception was still correlated with both left and right amygdala GMV (Left: *r* = 0.20, *p*<0.001; Right: *r* = 0.14, *p* = 0.03) after regressing out participants' IQ scores, suggesting that the association was specific to interval perception.

## Discussion

In the present study, we used the individual differences approach to investigate the neuroanatomical correlates between amygdala GMV and interval perception. As expected, we found significant positive correlations for the temporal cortex bilaterally, which extended to the superior temporal gyrus, middle temporal gyrus, planum polare, and Heschl's gyrus. Importantly, the GMV of both the left and right amygdala was correlated with interval perception, indicating that individuals with a larger amygdala GMV possess better ability in interval perception. Taken together, the present study provided one of the first neuroanatomical evidence that interval perception is not only related to the GMV of the auditory cortex but also the GMV of the amygdala, a region that is associated with emotional processing.

The finding that the GMV of the temporal cortex is related to interval perception is consistent with previous structural and functional MRI studies [14]–[16], [19], [52]–[57]. For instance, previous functional MRI studies have demonstrated that the discrimination of the pitch of a single note in the melody activates expansive regions in the temporal cortex, including the superior and middle temporal gyri bilaterally and the polanum polare [52], [54], [57]. Structural MRI studies that compared nonmusicians with musicians or participants suffering amusia with normal participants have also provided evidence linking the GMV of the superior temporal cortex and Heschl's gyrus to music processing [15], [18]–[20]. Importantly, unlike previous structural MRI studies focusing on the difference between groups, here we used the individual differences approach (see also [16]) to show that the variance of interval perception among individuals was partly accounted for by the variance of the GMV of the temporal cortex. Taken together, our study provides empirical evidence supporting Koelsch's music processing model [58], where melodic intervals are analyzed mainly in both the anterior and posterior parts of the temporal cortex.

The most important finding in the present study is, on a neuroanatomical level, that amygdala GMV was correlated with one's ability to perceive melodic intervals, suggesting that the amygdala may serve as a neural basis that links music and emotions. Besides previous functional neuroimaging studies that reveal amygdala activation during music processing [8]–[14], several neuropsychological studies suggest that the amygdala is necessary for the emotional processing of music. Indeed, patients with amygdala resections show impaired emotional responses to music, such as the impaired recognition of fearful or sad music [59], [60], and the loss of intensely pleasurable experiences while listening to pleasant music [61]. Other evidence suggesting the role of the amygdala in emotional processing of music comes from studies showing a direct anatomical connection between the auditory cortex and the amygdala, which allows the amygdala to respond to sound, even during sleep [62]–[64].

The link between interval perception and amygdala GMV may be a result of the fact that interval perception is associated with daily emotional experiences. However, it remains unclear why and how interval perception is correlated with emotional experiences. One possibility is that unexpected musical features, such as unexpected chords, are associated with less pleasant emotions than expected features [14], because people listening to music often form expectations as to what will happen next, and, depending on whether the expectations are fulfilled or not, people feel relaxed or tense, respectively [65]. Therefore, the dissonance caused by interval deviations, which are unexpected musical features, likely evoke unpleasant emotions in individuals. That is, individuals who have better interval perception would more often detect interval deviations and thereby experience stronger and more diverse emotions. To examine this possibility, future work is needed to characterize the link between music and emotion at the perceptual level.

In conclusion, our study demonstrates a possible neural basis underlying the link between music and emotion. However, it is unclear whether performance on other tasks related to one's music ability (e.g., rhythm perception) is also associated with amygdala GMV. Identification of the specific music components that are associated with activation of the amygdala may help in understanding exactly how music evokes emotion. Future studies are challenged to provide a more comprehensive picture of exactly how the amygdala is related to the broader concept of music.

## Supporting Information

Table S1 **The trial number in the interval test of MBEA and accuracy of the trials included in the modified interval test.**
(DOCX)
