# Supplementary material for: The Gray Matter Volume of the Amygdala Is Correlated with the Perception of Melodic Intervals: A Voxel-Based Morphometry Study
Source: PLoS One. 2014 Jun 12;9(6):e99889. doi: 10.1371/journal.pone.0099889 (PMC4055734; doi:10.1371/journal.pone.0099889)
Supplement: Table S1 — The trial number in the interval test of MBEA and accuracy of the trials included in the modified interval test. (DOCX) [file pone.0099889.s001.docx]

**Supporting Information**

**Table S1. The trial number in the interval test of MBEA and accuracy of the trials included in the modified interval test.**

| Interval | | |
| --- | --- | --- |
| Trial Number | Accuracy | Response Type |
| 1 | 0.83 | same |
| 2 | 0.68 | different |
| 4 | 0.68 | different |
| 7 | 0.83 | same |
| 10 | 0.85 | same |
| 11 | 0.70 | different |
| 13 | 0.70 | different |
| 14 | 0.88 | same |
| 16 | 0.63 | same |
| 18 | 0.83 | different |
| 19 | 0.75 | different |
| 21 | 0.88 | same |
| 22 | 0.80 | different |
| 23 | 0.75 | same |
| 25 | 0.88 | same |
| 26 | 0.83 | different |
| 27 | 0.65 | same |
| 29 | 0.88 | same |
| 30 | 0.70 | different |
| 31 | 0.70 | different |
